# Cerebral blood flow and cognitive outcome after pediatric stroke in the middle cerebral artery

**DOI:** 10.1038/s41598-021-98309-w

**Published:** 2021-09-30

**Authors:** Leonie Steiner, Andrea Federspiel, Jasmine Jaros, Nedelina Slavova, Roland Wiest, Maja Steinlin, Sebastian Grunt, Regula Everts

**Affiliations:** 1 Division of Neuropediatrics, Development and Rehabilitation, Department of Pediatrics, Inselspital, Bern University Hospital, University of Bern, Bern, Switzerland; 2 Psychiatric Neuroimaging Unit, Translational Research Center, University Hospital of Psychiatry, University of Bern, Bern, Switzerland; 3 Department of Interventional, Pediatric and Diagnostic Radiology, Inselspital, University Hospital, and University of Bern, Bern, Switzerland; 4 Institute of Diagnostic and Interventional Neuroradiology, Inselspital, Bern University Hospital, Bern, Switzerland; 5 Department of Diabetes, Endocrinology, Nutritional Medicine and Metabolism, Inselspital, Bern University Hospital and University of Bern, Bern, Switzerland

**Keywords:** Paediatric research, Stroke

## Abstract

Adaptive recovery of cerebral perfusion after pediatric arterial ischemic stroke (AIS) is sought to be crucial for sustainable rehabilitation of cognitive functions. We therefore examined cerebral blood flow (CBF) in the chronic stage after stroke and its association with cognitive outcome in patients after pediatric AIS. This cross-sectional study investigated CBF and cognitive functions in 14 patients (age 13.5 ± 4.4 years) after pediatric AIS in the middle cerebral artery (time since AIS was at least 2 years prior to assessment) when compared with 36 healthy controls (aged 13.8 ± 4.3 years). Cognitive functions were assessed with neuropsychological tests, CBF was measured with arterial spin labeled imaging in the anterior, middle, and posterior cerebral artery (ACA, MCA, PCA). Patients had significantly lower IQ scores and poorer cognitive functions compared to healthy controls (p < 0.026) but mean performance was within the normal range in all cognitive domains. Arterial spin labeled imaging revealed significantly lower CBF in the ipsilesional MCA and PCA in patients compared to healthy controls. Further, we found significantly higher interhemispheric perfusion imbalance in the MCA in patients compared to controls. Higher interhemispheric perfusion imbalance in the MCA was significantly associated with lower working memory performance. Our findings revealed that even years after a pediatric stroke in the MCA, reduced ipsilesional cerebral blood flow occurs in the MCA and PCA and that interhemispheric imbalance is associated with cognitive performance. Thus, our data suggest that cerebral hypoperfusion might underlie some of the variability observed in long-term outcome after pediatric stroke.

## Introduction

Arterial ischemic stroke (AIS) in children is a rare but devastating condition. The incidence of childhood stroke ranges from 1.3–13 per 100,000 children per year^1^, and boys are twice as likely to be affected as girls^2^. The etiology, presentation, and prognosis of stroke in children differ from those in adults^3^ and, although specific risk factors have been identified, the etiology of pediatric stroke often remains unclear^4^. Survivors of childhood stroke suffer from motor^5^ and cognitive restrictions^6–9^. While general intelligence is often within the average range^7,9–11^, deficits in specific cognitive subdomains can lead to learning difficulties and behavioral abnormalities^12^.

The severity of cognitive deficits are thought to vary according to lesion-related characteristics, such as size and location, as well as alterations in functional networks and cerebral blood flow. Therefore, it is important to study cerebral, post-ischemic changes in detail. A modern approach to investigate cerebral blood flow is by means of arterial spin labeling (ASL). This is a non-invasive, non-ionizing magnetic resonance imaging (MRI) technique using arterial blood as an endogenous tracer. ASL provides a safe, economical, and quantifiable measure of cerebral blood flow reflecting the level of glucose metabolism associated with neuronal activity^13^. AIS is associated with an acute local reduction of cerebral blood flow^14^. In adults under normal conditions, a cerebral blood flow of 50–80 ml/100 g/min ensures a sufficient energy supply to the brain^15^, however, a short-term reduction to < 20 ml/100 g/min leads to reversible nerve cell damage and a reduction to < 15 ml/100 g/min leads to necrosis of the brain parenchyma within a few minutes^16^. As cerebral blood flow mirrors the brain’s metabolic demands and neuronal activity, its measurement provides important information on brain activity and functional recovery^17^.

Both, hyper- and hypoperfusion of cerebral blood flow have been found after AIS in the acute and sub-acute phase^17–20^. Large lesions and more severe intracranial arteriopathy have been associated with hypoperfusion, whereas smaller lesions have been associated with reperfusion or hyperperfusion^17,18^. Hyperperfusion is thought to be due to neuronal hyperexcitability following the insult or due to stroke-associated seizures. Hypoperfusion has previously been suggested to resolve over time in accordance with early behavioral recovery patterns^21–23^. However, case studies of patients in the chronic phase after stroke report hypoperfusion in the lesioned hemisphere^22,24,25^ and relate it to functional deficits, i.e. language problems^22^, reading deficits^24,26^, and phonological competence^26^.

In the chronic stage after stroke, reductions of ipsilesional cerebral blood flow was correlated with infarct size in patients after left-hemisphere stroke, indicating sustained hypoperfusion in the affected hemisphere^20^. Compared to acute stroke^27^, the understanding of cerebral perfusion in the chronic stage after stroke is far less clear^20,24,25,28^ and was barely described in children and adolescence so far^17,20,25^.

Besides measuring cerebral blood flow after AIS, it is crucial to study the interhemispheric imbalance between ipsilesional and contralesional perfusion. A longitudinal study with adults in the subacute phase after AIS showed that sustained hemispheric perfusion imbalance is associated with poor motor function, suggesting that the interhemispheric balance may be critical for motor recovery after AIS^29^. In line with this finding, we observed in a previous study that patients with hemiparesis after pediatric AIS present with sustained interhemispheric perfusion imbalance, which was related to poorer manual ability^30^. So far, however, the relationship between cerebral blood flow and cognitive outcome has rarely been studied.

Depending on the region of interest, positive as well as negative correlations occurred between cerebral blood flow and cognitive functions such as i.e. intelligence quotient (IQ) in 39 healthy children aged 7–17 years^31^. A longitudinal study in a healthy cohort of older adults showed that cerebral blood flow can predict both, general cognitive ability as well as specific cognitive functions, with higher blood flow enabling better cognitive functions^32^.

There is no clear relationship between cerebral blood flow and cognitive functions in patients^33^. A study using SPECT in 21 pediatric patients with moyamoya disease (aged 5–14 years) showed a significant positive association between regional cerebral blood flow and intelligence, perceptual reasoning and processing speed^34^. Contrary, a negative correlation between cerebral blood flow (measured with ASL) and IQ has been found in 24 children with sickle cell anemia (aged 6–12 years^33^). In line with the negative correlation between cerebral blood flow and cognition are the results of a study on 26 children with hypoxic-ischemic encephalopathy. Neonatal cerebral blood flow assessed using PET correlated negatively with IQ measured in childhood^35^.

Results of the different clinical studies may not be comparable due to differences in perfusion assessments, cognitive assessment, age at assessment and underlying disease. Cerebral blood flow in the chronic phase after pediatric AIS has never been investigated so far and the relationship between interhemispheric perfusion balance and cognitive outcome has not yet been studied, neither in healthy subjects nor in patients following AIS.

Therefore, the aim of the present study was to investigate cerebral perfusion in patients in the chronic stage after pediatric arterial ischemic stroke in the MCA compared to healthy controls. Further, we aimed to investigate the relationship between interhemispheric perfusion balance and long-term cognitive outcome in children after AIS. In accordance with the existing literature, we hypothesized that (1) children after AIS in the MCA have lower cerebral blood flow in the ipsilesional hemisphere compared to healthy controls even years after stroke and (2) sustained interhemispheric cerebral blood flow imbalance is associated with lower cognitive outcome such as previously described for motor outcome^29,30^. Disentangling the relationship between cerebral blood flow and cognitive functions after AIS will indicate whether cerebral blood flow can be used as a proxy for rehabilitation capacity.

## Methods

We report on data from the HERO Study^36^ examining functional reorganization after childhood stroke with a cross-sectional as well as a longitudinal approach. The HERO Study was approved by the local ethics committee of the Canton of Berne (KEK 212/13) and the ethics committee of the Children’s University Hospital and was performed in accordance with the declaration of Helsinki. All participants, or their parent or legal guardian if they were younger than 18 years, gave written informed consent prior to enrollment. Participants were compensated for their participation (with a movie voucher or book voucher).

### Participants

Patients were identified by the Swiss Neuropediatric Stroke Registry (SNPSR)—a multicenter, prospective, and population-based registry that includes children diagnosed with AIS under the age of 16 years^5^. Patients were included if AIS had occurred at least 2 years prior to the assessment. Exclusion criteria were active epilepsy, iron implants, claustrophobia and behavioral problems that make an MRI scan impossible.

Of the twenty nine patients recruited for the HERO Study, 14 had an arterial ischemic stroke in the MCA territory after exclusion of patients due to developmental delay or behavioral problems that interfered with compliance (n = 2), bilateral lesions (n = 4), retainer artifacts (n = 1), error in T1-weighted anatomical image or ASL sequences (n = 2) or neonatal stroke (n = 3). Healthy controls met the following inclusion criteria: absence of neurological disease or psychiatric disorders, no deficit in general intellectual functioning as measured with IQ (IQ > 85), and no contraindications for MRI (metal braces, metallic implants). Of the forty four healthy controls eight had to be excluded because of incorrect relaxation time in ASL sequence (n = 2), retainer artifacts (n = 2), missing age norms for the youngest children (< 7 years, n = 4). Detailed clinical characteristics of the study participants are provided in Supplementary Table S1.

### Cognitive outcome

All tests were conducted by a trained neuropsychologist. To obtain a reliable and valid assessment of different cognitive domains, an extended and standardized test battery was adopted. Details on the tests have been previously published (HERO Study^36^). Raw scores for all tests were transformed into age-dependent standard scores (M = 100, SD = 15) according to the relevant test manual. Test scores measuring the same cognitive domain were *z*-transformed and the means from the tasks were calculated to obtain the domain-specific index. IQ was measured using the Test of Nonverbal Intelligence (TONI-4)^37^, which is a language-free test assessing fluid intelligence in children and adults.

#### Executive functions

Verbal working memory was assessed using the subtests Letter-Number-Sequencing of the Wechsler Intelligence Scale for Children (WISC-IV)^38^ or the Wechsler Intelligence Scale for Adults (WAIS-IV)^39^ depending on the age of the participant. Visuo-spatial working memory was assessed using the spatial positioning subtest of the Learning and Memory Test (basic-MLT). Inhibition was measured using the Go/NoGo task of the Test of Attentional Performance (TAP)^40^ and the Color Word Interference Test (CWI) 3rd condition of the Delis-Kaplan Executive Function System™ (D-KEFS™^41^. For the assessment of shifting, the Trail-Making-Test and the 4th condition and the CWI of the D-KEFS™^41^ were used.

#### Processing speed

Processing speed was measured with the subtests Symbol Search and Digit Symbol-Coding of the WISC-IV^38^ or the WAIS-IV^39^ depending on the age of the participant.

#### Attention

Selective attention was evaluated with the cancellation task of the WISC-IV^38^ or the WAIS-IV^39^. The Divided Attention task of the TAP^40^ was also used.

#### Memory

Verbal learning was assessed with a standardized multitrial learning task consisting of five repeated auditory presentations of a 15-word list that had to be recalled by the participant immediately after each presentation (VLMT)^42^. Visual learning was measured with the Rey Visual Design Learning Test (RVDLT). This test consists of 15 cards displaying simple geometric forms that are presented to the child one by one, with an interval of 2 s per card. After all test items have been shown, the child is asked to draw as many of the items as she or he can recall. This procedure is repeated another four times (learning and recall phase).

#### Visuo-spatial abilities

Visuo-spatial abilities were measured with the Beery-Buktenica Developmental Test of Visual-Motor Integration (VMI), which is a standardized copy forms-type test used to assess visual-motor integration. The three subtests: (visual-motor integration subtest, test of visual perception, and test of motor coordination were individually administered in that order to each participant as described in the VMI Administration, Scoring, and Teaching Manual (4th edition). Each perceptual test was scored according to the published instructions^43^.

#### Overall cognitive outcome

The overall cognitive outcome score was calculated as the mean of all domain-specific index scores. All cognitive domain scores were *z*-transformed and summarized.

### Neuroimaging

#### Structural imaging

High-resolution anatomical T1-weighted images were acquired on a 3 T Magnetom Verio Siemens scanner (Siemens, Erlangen, Germany) using a magnetization-prepared rapid acquisition gradient-echo (MP-RAGE) sequence (repetition time = 2530 ms; echo time = 2.92 ms; inversion time = 1100 ms; 160 sagittal slices; flip angle = 9°; field of view = 256 mm × 256 mm; matrix dimension = 256 × 256; isotropic voxel resolution = 1 mm^3^). The scan duration was 5 min 05 s.

Lesion-related characteristics were determined by a board-certified neuroradiologist. Ischemic lesions were manually traced to calculate the volume of affected brain tissue. Lesion size was defined as the affected brain tissue in relation to the total brain volume (ratio). Total intracranial volume (gray matter (GM), white matter and cerebrospinal fluid (CSF)) was calculated using the MATLAB-based toolbox SPM (SPM12, Wellcome Department of Imaging Neuroscience, London, England). Lesion laterality was classified depending on the affected hemisphere (i.e. left, right, or bilateral) and lesion location was divided into three categories (cortical, subcortical, combined cortical and subcortical, according to Everts et al. 2008). All lesions were flipped to the left hemisphere, so that the left hemisphere was always the ipsilesional hemisphere. Hence, in controls, the left hemisphere was compared to the ipsilesional hemisphere in patients.

#### Arterial spin labeling

To assess cerebral blood flow, we adopted a pseudo-continuous arterial spin labeling (pCASL) sequence^44,45^. Specifically, an alternating sequence of label and control images was acquired and labeling was performed at 80 mm below the isocenter of the imaging region. A post-labeling delay (PLD) of 1.25 s was set with a label time of 1.6 s. A total of 16 slices with a slice thickness of 6 mm were recorded sequentially from inferior to superior. Each pCASL measurement was repeated 120 times. Images were acquired using the following parameters: TE = 12 ms; TR = 3400 ms; field of view, 230 mm^2^; matrix size, 64 × 64; flip angle 90°; voxel size, 3.6 × 3.6 × 6.0 mm. Additionally, one M0 image for tissue at equilibrium magnetization was recorded with TR = 8000 ms and PLD = 5000 ms. All other parameters were unchanged. The duration of the ASL scan was 6 min 58 s.

SPM12 and MATLAB (MathWorks Inc.; version R2017a) was used for all processing steps. ASL time series were realigned to correct for motion artifacts and anatomical T1 images were segmented into GM, white matter, and CSF. The estimation of cerebral blood flow can be performed with the ASL technique. In fact, a calibrated cerebral blood flow measure can be obtained using a one-compartment model^46,47^ solving the following equation:

$$CBF = \left( {\frac{\lambda \cdot \Delta M}{{2 \cdot \alpha \cdot M_{0} \cdot T_{1b} }}} \right) \cdot \left( {\frac{1}{{e^{{ - {w \mathord{\left/ {\vphantom {w {T_{1b} }}} \right. \kern-\nulldelimiterspace} {T_{1b} }}}} - e^{{ - {{(\tau + w)} \mathord{\left/ {\vphantom {{(\tau + w)} {T_{1b} }}} \right. \kern-\nulldelimiterspace} {T_{1b} }})}} }}} \right).$$

The variables are as follows: post-labeling delay (ω) (PLD), labeling duration (τ), blood/tissue water partition coefficient λ = 0.9 g/mL, and labeling efficiency α = 0.85^21^. In the human brain, and for 3.0 T, a decay time for labeled blood T1b = 1650 ms is assumed. Moreover, M0 are the equilibrium brain tissue magnetization images^13,46,48^ and were acquired in separate runs. ΔΜ represents the time series obtained by subtraction of control and label images. The ASL images used for cerebral blood flow quantification were all recorded and processed according the “ASL white paper”^49^. All MRI modalities were processed so that a normalized standard space (Montreal Neurological Institute coordinate system, MNI) was available to ensure the extraction of cerebral blood flow values for homologous brain regions. Each cerebral blood flow map was then masked with the segmented GM anatomical images. We used a threshold of 0.7 for the creation of each GM mask, which was then applied to each cerebral blood flow map.

To ensure that cerebral blood flow was only measured in anatomically intact tissue, we superimposed the lesion masks generated from the T1-weighted anatomical images on the cerebral blood flow map. The resulting mean cerebral blood flow maps were then co-registered to the anatomical scans, normalized to the MNI and spatially smoothed with a Gaussian kernel (8 mm, full-width at half-maximum). Cerebral blood flow was measured throughout the brain and separately in each of the hemispheres in the territories of the anterior (ACA), middle (MCA) and posterior cerebral artery (PCA).

Cerebral blood flow balance was assessed by calculating cerebral blood flow difference scores between the ipsilesional and contralesional cerebral blood flow of the ACA, MCA and PCA.

To control for subject motion, deviations from the initial position were assessed during the ASL scan. Deviations were measured along the x-, y- and z-axes in mm (x, y, z) and in radians (α, β, γ).

### Statistical analysis

All analyses were performed using the statistical software package R 3.6.0 (Core Team, 2019). Variables were tested for normality with the Shapiro–Wilk test. Mean values between two groups were compared using one-sided (for cognition) or two-sided (for cerebral blood flow) independent samples *t* tests (normally distributed variables) or Mann–Whitney *U* tests. For correlation analyses, Pearson (normally distributed variables) or Spearman correlations (non-normally distributed variables) were applied. To investigate the relationship between cerebral blood flow and cognition, we applied partial correlations (Spearman), with lesion size as covariates. To account for the effects of multiple hypothesis testing (type I error), false discovery rate (FDR) correction was employed for all analysis. Results of *P* < 0.05 FDR-corrected were considered significant.

## Results

### Demographics

Patients and healthy controls were comparable in terms of sex (*χ*^2^ = 0.828, *p* = 0.363) and age at examination (*t* = 2.226, *p* = 0.822). Mean age at stroke was 6.2 years (SD = 3.8, range = 1.17–14.33), mean time since stroke was 7.2 years (SD = 3.9, range = 2.1–15.5). Mean lesion size corrected for intracranial volume was 1.8 mm^3^ (SD = 3.1, range = 0.003–11.7 mm^3^). Of the AIS group, 78.6% (*n* = 11) had a lesion in the left and 21.4% (*n* = 3) in the right hemisphere. A subcortical lesion was seen in 71.4% (*n* = 10) of the patients and 28.6% (*n* = 4) had a combined lesion (subcortical and cortical). No patient had an exclusively cortical lesion. Detailed clinical characteristics of the study participants are provided in Supplementary Table S1.

### Cognitive outcome

Patients mean cognitive performance was within the normal range in all cognitive domains. However, when compared to healthy controls, patients had significantly reduced overall cognitive functions (*U*(2) = 76.0, *p* = 0.001). In particular, IQ (*U*(2) = 115, *p* = 0.003), memory (verbal and visual learning) (*U*(2) = 112.0, *p* = 0.003), working memory (*U*(2) = 118, *p* = 0.005), cognitive flexibility (U(2) = 159.5, *p* = 0.026), attention (U(2) = 151.5, *p* = 0.019), processing speed (*U*(2) = 133.5, *p* = 0.007), and visuo-spatial abilities (*U*(2) = 134, *p* = 0.007) differed significantly between the AIS group and healthy controls (Table 1). There was no significant between group difference for inhibition, even though the AIS group displayed worse mean performance in this domain than controls. On a descriptive level, patients with combined lesions (n = 4) showed worse median cognitive performance across all cognitive domains when compared to patients with subcortical lesions only (n = 10, see Fig. S1).

**Table 1:** Cognitive performance in patients and controls.

	Patients n = 14 *Md* (*SD*)	Controls n = 36 *Md* (*SD*)	*U*	*P*
**IQ**	92.5 (9.12)	101 (10.14)	115.0	0.003*^†^
range	84 to 120	89 to 127		
**Memory (visual/verbal learning)**	− 0.51 (0.91)	0.29 (0.70)	112.0	0.003*^†^
range	− 1.89 to 0.67	− 1.88 to 0.92		
**Working memory**	− 0.55 (0.98)	0.22 (0.63)	118.0	0.005*^†^
range	− 2.19 to 0.85	− 1.01 to 1.59		
**Inhibition**	− 0.31 (0.87)	− 0.04 (0.64)	213.0	0.206
range	− 2.05 to 1.15	− 1.46 to 1.08		
**Cognitive flexibility**	− 0.45 (0.65)	0.16 (0.63)	159.5	0.026*^†^
range	− 1.76 to 0.63	− 1.44 to 0.95		
**Attention**	− 0.44 (0.69)	0.37 (0.61)	151.5	0.019*^†^
range	− 1.17 to 0.75	− 0.46 to 0.93		
**Processing speed**	− 0.42 (1.09)	0.33 (0.91)	133.5	0.007*^†^
range	− 2.65 to 0.91	− 1.74 to 1.82		
**Visuo-spatial abilities**	− 0.77 (1.02)	0.35 (1.19)	134.0	0.007*^†^
range	− 1.70 to 1.88	− 1.07 to 3.44		

*^†^*p* < 0.05, after FDR correction. *Md* = median, *SD* = standard deviation.

Lesion size correlated negatively with cognitive variables (Table 2), indicating that patients with larger stroke volume performed worse in almost all cognitive domains (r = − 0.470 to r = − 0.756). No significant association was found between cognitive variables and time since stroke and age at stroke (*p* > 0.05).

**Table 2:** Relation between stroke characteristics and cognition.

	Inhibition	Cognitive flexibility	Working memory	Processing speed	Attention	Visuo- spatial abilities	Memory
Age at AIS	0.345	− 0.046	− 0.046	0.095	0.130	− 0.292	0.253
Time since AIS	− 0.068	0.160	0.367	− 0.009	− 0.130	− 0.262	0.429
Lesion size	− 0.486*	− 0.437	− 0.459*	− 0.700*^†^	− 0.508*	− 0.503*	− 0.235

*^†^*p* < 0.05; significant after FDR correction.

### Cerebral blood flow

To ensure that differences in cerebral blood flow were not related to motion during MR scanning, motion parameters were compared between patients and healthy controls. No significant differences were found between patients and controls. Detailed results with *z* and *p* values are provided in Supplementary Table S2.

In all vessel territories median cerebral blood flow of patients was lower than in controls (Table 3) with significant cerebral blood flow differences ocurring in the ipsilesional MCA and PCA (MCA *U*(2) = 121, *p* = 0.004; PCA (*U*(2) = 147, *p* = 0.011). Descriptively, median cerebral blood flow was slightly lower in patients with subcortical lesions (n = 10) when compared to patients with combined lesions (n = 4) in all vessel territories (see Fig. S2).

**Table 3:** Cerebral blood flow in patients after MCA stroke and controls.

	Patients n = 14 *Md* (*SD*)	Controls n = 36 *Md* (*SD*)	*U*	*P*	*Cohens d*
**ACA**					
Ipsilesional/left	49.83 (13.22)	57.71 (12.30)	180.0	0.062	0.45
Contralesional/right	51.58 (12.52)	59.73 (12.92)	212.0	0.199	0.26
Perfusion imbalance	2.75 (5.41)	1.83 (2.85)	203.0	0.149	0.30
**MCA**					
Ipsilesional/left	42.07 (14.80)	52.92 (10.41)	121.0	0.004*^†^	0.87
Contralesional/right	49.59 (12.04)	52.82 (9.96)	225.0	0.286	0.17
Perfusion imbalance	4.15 (14.36)	2.47 (2.39)	155.0	0.018*^†^	0.62
**PCA**					
Ipsilesional/left	38.45 (13.63)	45.88 (11.96)	147.0	0.011*^†^	0.68
Contralesional/right	45.80 (14.10)	51.11 (12.34)	176.0	0.052	0.48
Perfusion imbalance	5.89 (6.93)	4.86 (2.89)	221.0	0.257	0.19

*ACA* anterior cerebral artery, *MCA* middle cerebral artery, *PCA* posterior cerebral artery, *Md* median. Note that in controls, the left hemisphere corresponds to the ipsilesional hemisphere in patients (all lesions were flipped to the left hemisphere). The right hemisphere in controls corresponds to the contralesional hemisphere in patients. *p < 0.05, uncorrected, *^†^*p* < 0.05, after FDR correction for multiple comparisons.

**Figure 1:**
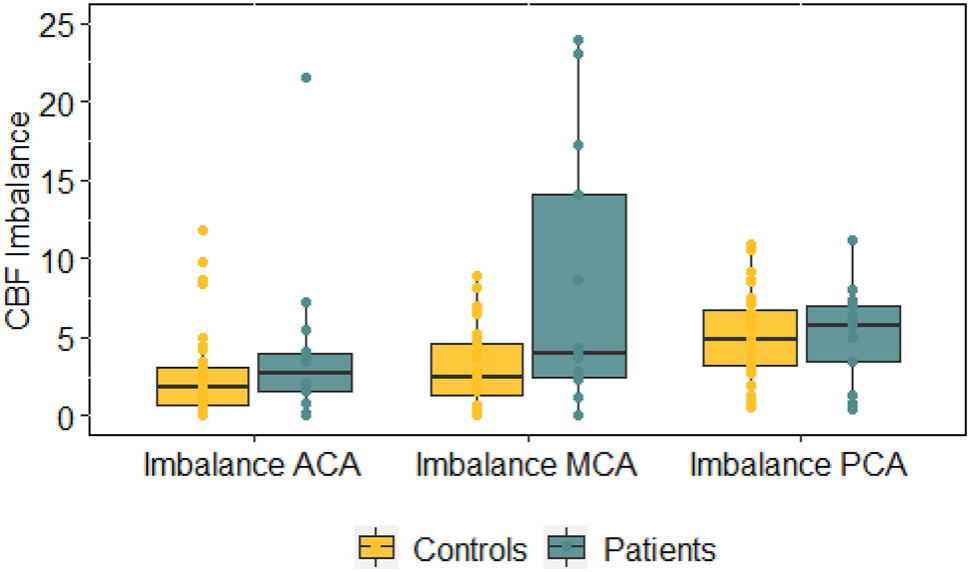
Cerebral blood flow imbalance in patients and controls. Boxplot show median, interquartile range, minimum and maximum score and extreme values.

As hypothesized, cerebral blood flow imbalance in the MCA (calculated as difference score between the ipsilesional and contralesional cerebral blood flow) was significantly higher in patients than controls (Table 3; Fig. 1). Whereas median cerebral blood flow imbalance was higher in patients across all vessel territories, there were no significant group differences for cerebral blood flow imbalance in the ACA and PCA.

### Association between cerebral blood flow imbalance, lesion size and cognitive outcome

First, we analyzed the association between cerebral blood flow imbalance and lesion related variables and cognitive outcome (Table 4). Analyses revealed no significant associations between cerebral blood flow imbalance and age at stroke nor with time since stroke (*p* < 0.05). However, lesion size correlated positively with cerebral blood flow imbalance in the MCA (r = 0.695, *p* = 0.036). Patients with combined lesions (larger lesions, n = 4) showed higher median cerebral blood flow imbalance than patients with subcortical lesions (smaller lesions, n = 10) when looking at the data descriptively (see Fig. S3).

**Table 4:** Correlation between cerebral blood flow imbalance and cognition in patients (controlled for lesion size).

	MCA imbalance	ACA imbalance	PCA imbalance
	Patients *r*	Patients *r*	Patients *r*
Inhibition	0.095	− 0.202	− 0.103
Cognitive flexibility	− 0.531*	− 0.571*	− 0.259
Working memory	− 0.787*^†^	− 0.302	− 0.147
Processing speed	0.051	0.136	− 0.239
Attention	0.233	− 0.255	− 0.093
Visuo-spatial abilities	0.030	− 0.084	0.254
Memory	− 0.364	− 0.310	− 0.068

*r* correlation coefficients, *p < 0.05, uncorrected, *^†^*p* < 0.05, after FDR correction for multiple comparisons. *MCA* middle cerebral artery, *ACA* anterior cerebral artery, *PCA* posterior cerebral artery.

In patients, partial correlations (with lesion size as covariate) revealed significant negative relationships between interhemispheric cerebral blood flow imbalance in the MCA and working memory (*r* = − 0.787, *p* = 0.005).

## Discussion

This cross-sectional study adopted arterial spin labeled perfusion imaging to investigate cerebral blood flow after pediatric AIS in the MCA and its relation to long-term cognitive outcome in the chronic phase years after stroke. The performance of patients was significantly worse than that of controls in several cognitive domains providing further support for cognitive deficits after pediatric AIS such as presented in previous studies^8,10,12^. We found significantly lower cerebral blood flow in the ipsilesional MCA and PCA in patients than in controls. Imbalance of cerebral blood flow in the MCA did significantly differ between groups. According to our second hypothesis, we found that sustained hemispheric imbalance of cerebral blood flow was negatively associated with working memory.

Our finding of reduced mean ipsilesional cerebral blood flow after pediatric AIS across all vessel territories (reaching significance for the MCA and PCA) is in line with findings from studies of adult patients with chronic stroke^20^ or in the subacute phase after stroke^29^, indicating sustained hypoperfusion in the affected hemisphere^20^. The reduction of cerebral blood flow in ipsi- and to some degree also in contralesional vessel territories, even several years post-stroke, suggests that cerebral blood flow perfusion has not fully recovered. This finding is in line with studies showing that adult patients after stroke show decreased cerebral blood flow due to impaired autoregulation in the long term^49–52^. Our data follows up on a case study^24^ suggesting that even in structurally intact brain areas, cerebral perfusion is altered in the long-term. A stroke causes hypoperfusion to the stroke core where neurons are likely to die^53^. Hypoperfusion additionally occurs in peri-infarct tissue where neurons may remain alive if blood flow is restored within two days after stroke^54^. However, hypoperfusion in the peri-infarct area can remain for weeks after stroke^22^. In line with the results of the present study, the effects of stroke can also affect remote functional networks^26^, likely due to diaschisis (electrical, metabolic, or blood flow dysfunction in functionally connected areas remote from the lesion^55^) or due to other signals leading to a widespread disconnection of brain networks^54^. In adult patients after stroke, hypoperfusion of anatomically intact areas can be seen up to a year after stroke^56^. Hence, functional brain recovery is suggested to crucially depend on what happens in the peri-infarct areas and in larger functional neural network^57^.

Our results are supposedly contrasting previous findings on the association between cerebral blood flow and lesion size^17^. On a descriptive level, our data shows higher cerebral blood flow in patients with combined lesions (larger lesions n = 4, mean age at assessment 15.3 years) than patients with subcortical lesions only (smaller lesions, n = 10, mean age at assessment 12.7 years). In a previous study, hyperperfusion occurred more likely in children with smaller stroke volume whereas hypoperfusion related to larger lesions in the acute and subacute phase after stroke^17^. This opposing finding is likely explained by differences in respect to the age at assessment (^17^: 9.2 years) and the time-point of the follow-up assessment (^17^: average of 6 months after stroke; our patients: average of 8.4 years for patients with combined lesions and 6.7 years after stroke for patients with subcortical lesions; see Table S1). Cerebral blood flow characteristics are known to change with age^31^ and during recovery^22,56^ which hinders a comparison of studies with different patient characteristics.

Further, the literature shows that cerebral blood flow imbalance is more accentuated in children with severe arteriopathy when compared to mild arteriopathy^18^, a finding which is consistent with the results of the present study, where larger lesions related to higher imbalance. In adults with arterial disease, the extent of white matter lesion modified the association between cerebral blood flow and executive functions; the association between lower cerebral blood flow and worse executive functions became stronger with increasing volumes of white matter lesion^50^. Consequently, patients with large lesions may suffer from a “double-hazard” phenomenon: larger lesions correlated with lower cerebral blood flow in the affected hemisphere and both aspects are likely affecting cognitive outcome negatively.

Decreased cerebral blood flow is thought to be associated with reduced functional and structural reorganization capacities, which in turn might lead to slower cognitive recovery. During childhood, brain development undergoes shifts in functional connectivity^58,59^, hemodynamic properties^60^, cortical surface expansion^61^, increases in white matter volume^62^ and decrease in synapse density due to pruning^63^. Cerebral blood flow plays an important role during these changes, as it supplies blood and nutrients to the brain, supports ongoing development and likely reflects decreased synaptic density^63,64^.

Cerebral blood flow imbalance (calculated as difference score between the ipsilesional and contralesional cerebral blood flow) differed significantly in the MCA between healthy controls and the AIS group. Cerebral blood flow imbalance of the MCA was negatively associated with working memory performance in children after pediatric AIS. Working memory is a crucial functional domain that underlies many higher-order cognitive functions such as reading, arithmetics and self-regulation processes^65^. Mean working memory performance was 0.55 standard deviations below the mean in the present patient sample and presents the most pronounced deficit among the cognitive functions measured. However, cognitive flexibility was also negatively related to cerebral blood flow imbalance of the MCA with weak to moderate effect sizes. Our findings suggest that a certain hemispheric imbalance seems to promote the persistence of cognitive deficits. Wiest et al.^29^ examined cerebral blood flow imbalance in adults in the subacute phase of AIS and reported similar results, showing that incomplete motor recovery was associated with a greater interhemispheric imbalance. After stroke, alterations in neurovascular function, such as cerebrovascular reactivity (CVR), might help to explain the present results. The co-occurrence of hypoperfusion and reduced CVR has been reported previously^66^ and reduced CVR has been reported in lesioned brain areas in both acute and chronic recovery^66,67^. Additionally, cerebral blood flow mirrors metabolic demand and neuronal activity. Thus, decreased ipsilesional cerebral blood flow may reflect decreased neuronal activity due to reductions in the neurons’ metabolic needs and network connectivity after the loss of cells within the lesioned brain area^20^. This would be in line with the negative correlation between cerebral blood flow and cognitive functions described previously^33^. Whether the relationship between cerebral blood flow imbalance and cognitive outcome is of causal nature remains to be determined in future studies using methods that have the power to unravel causality.

Overall, our data support the idea that cerebral hypoperfusion might underlie some of the variability observed in long-term outcome after stroke. The present findings offer insights into the state of cerebral perfusion years after stroke and highlight the role of interhemispheric perfusion balance in the MCA for working memory performance. Our results provide further support that the assessment of cerebral blood flow perfusion with ASL presents a possible index for evaluating the effectiveness of rehabilitation at the perfusion level.

### Strengths and limitations

This study had some notable strengths. First, ASL is gaining attention as a non-invasive alternative to invasive perfusion imaging after stoke and can be performed in 2–5 min. Second, an extended test battery was adopted to investigate several cognitive domains with different tasks to enable the assessment of domain-specific outcome, as well as an overall cognitive outcome score. Third, our study included a homogeneous sample of patients after pediatric AIS in the MCA and excluded children following neonatal arterial ischemic stroke, periventricular venous infarction or stroke to other vessel territories. This helps in disentangling the effects of pathophysiological mechanisms and lesion-related characteristics on cerebral perfusion alterations and on cognitive outcome.

Nevertheless, our study has some limitations. First, our results are based on a small and clinically heterogeneous sample. However, there is the chance of recruitment bias towards a rather homogeneous and well-functioning patient group, as patients with behavior problems or severe handicaps were unable to follow the study regime and hence were excluded. The study sample included children across a wide age range at time of assessment and hence at different neurodevelopmental stages. The rarity of childhood stroke (1–2 cases per center per year in Switzerland based on estimates from the SNPSR from 2000 to 2019) makes recruitment a challenge. Nonetheless, further research is needed to replicate our findings in a larger cohort and confirm that cerebral blood flow alterations are related to cognitive functions throughout post-stroke recovery. Secondly, AIS in childhood is based on multifactorial causes, which themselves can be associated with perfusion characteristics and cognitive outcome^68^.

## Conclusion

Our findings revealed that ipsilesional cerebral blood flow is reduced across all vessel territories even years after pediatric stroke. Hemispheric imbalance of the cerebral blood flow is negatively associated with cognitive outcome. This finding has important clinical and theoretical implications and may need to be taken into account when examining the relationship between brain lesion and cognition in the future. First, measurements of cerebral perfusion imbalance in the acute phase after stroke might be useful in predicting future cognitive impairment. Second, changes in cerebral blood flow imbalance may be used to track the process of recovery longitudinally, in particular as a marker of neuronal recovery. Identification of optimal treatment strategies to support recovery is still limited by the wide variance in outcomes of patients after AIS. Thus, identifying biomarkers that distinguish patient subgroups will help to identify factors that are important for successful recovery after pediatric AIS. The results of this study raise new questions for future research including whether rehabilitation efforts can increase interhemispheric perfusion recovery. In the future, a multimodal imaging approach will be needed to find out how functional networks measured with functional MRI or resting-state fMRI are related to cerebral blood flow.

## Supplementary Information

Supplementary Information.
