# Supplementary material for: Cerebral blood flow and cognitive outcome after pediatric stroke in the middle cerebral artery
Source: Sci Rep. 2021 Sep 30;11:19421. doi: 10.1038/s41598-021-98309-w (PMC8484584; doi:10.1038/s41598-021-98309-w)
Supplement: Supplementary file 1 — Supplementary Information. [file 41598_2021_98309_MOESM1_ESM.pdf]

Supplementary Material

Table S1. Lesion characteristics of individual participants.

No.	Sex	Age	Handedness	Age at stroke	Time since stroke	Lesion location	Lesion side	Lesion volume	Vessel territory	Etiology (CASCADE)	Specification of etiology	Initial PSOM	Chronic PSOM
1	1	6.08	Right	4.00	2.08	SC	Left	0.01	MCA/ICA	Arteriopathy	Preceeding varicella infection	1	0
2	1	15.5	Right	11.75	3.58	SC	Right	0.003	MCA	Arteriopathy	Vasculopathy most likely in the context of hashimoto thyroiditis	0.5	0
3	2	18.42	Left	6.25	12.08	SC+C	Left	1.08	MCA	Other	Unclear despite complete workup	4	1
4	2	8.33	Right	4.83	3.50	SC	Right	0.10	MCA	Other	Unclear despite complete workup	1	0
5	1	14.08	Right	3.42	10.50	SC	Right	0.35	MCA	Arteriopathy	Unclear despite complete workup	1	1
6	1	15.33	Right	7.83	7.41	SC	Left	0.02	MCA	Arteriopathy	Preceeding varicella infection	0	0
7	2	11.42	Left	3.5	7.83	SC	Left	0.01	MCA	Arteriopathy	Preceeding varicella infection	1	0.5
8	2	13.17	Right	5.75	7.33	SC+C	Left	3.47	MCA	Other	Unclear despite complete workup	2	0
9	2	18.75	Right	14.33	4.42	SC	Left	0.05	MCA/ICA	Arteriopathy	Unclear despite complete workup	1	0
10	2	8.75	Left	5.33	3.42	SC+C	Left	7.74	MCA	Other	Vasculopathy most likely in the context of systemic inflammation (toxic shock syndrome)	4	2
11	2	9.42	Left	1.5	7.92	SC	Left	1.00	MCA	Multi-factorial	Preceeding varicella infection	3	0.5
12	2	11.67	Right	6.67	4.92	SC	Left	0.01	MCA	Cardio-embolic	Patent foramen ovale, multiple atrial communications	0	0
13	1	20.75	Left	9.83	10.92	SC+C	Left	11.68	MCA	Other	Unclear despite complete workup	2	2.5
14	2	16.75	Left	1.17	15.50	SC	Left	0.09	MCA	Other	Unclear despite complete workup	0	0

Note. Calculation of lesion size ratio: volume of lesion/total intracranial volume × 100. SC, subcortical; C, cortical; SC + C, combined lesion; PSOM, pediatric stroke outcome measure; initial = 1 month after stroke; chronic = at the time of study assessments.

Table S2. Motion during measurement of cerebral blood flow.

	Mann-Whitney-U	z value	*p* value
X axis motion (mm)	337.00	−0.335	0.738
Y axis motion (mm)	320.00	−0.619	0.536
Z axis motion (mm)	271.00	−1.439	0.150
X axis angle motion (radians)	355.00	−0.033	0.973
Y axis angle motion y (radians)	348.50	−0.142	0.887
Z axis angle motion z (radians)	340.50	−0.276	0.782

Note. Deviations from the initial position were measured along the x-,y- and z-axes in mm (x,y, z) and in radians (α,β,γ).

Figure S1. Cognitive performance in patients with subcortical lesions (n = 10), patients with combined lesions (n = 4) and controls.

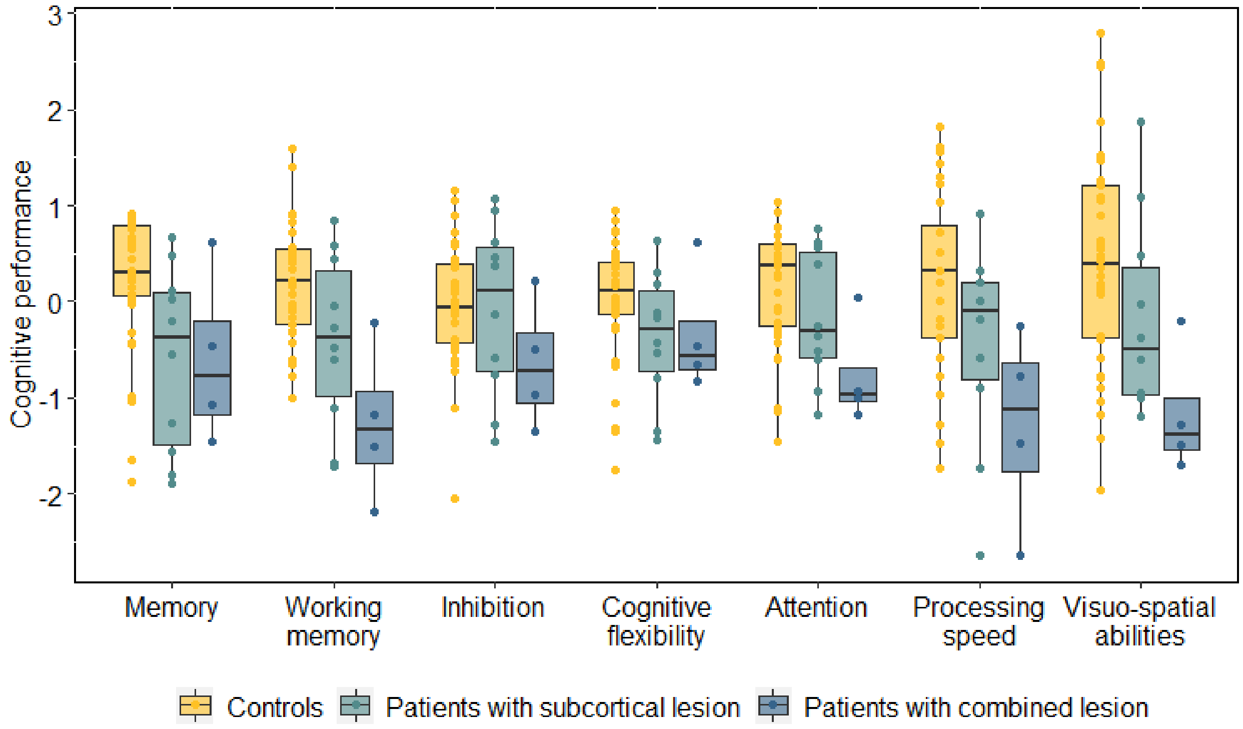

Note. Boxplots show median, interquartile range, minimum and maximum score and extreme values.

Figure S2. Cerebral blood flow in patients with subcortical lesions (n = 10), patients with combined lesions (n = 4) and controls.

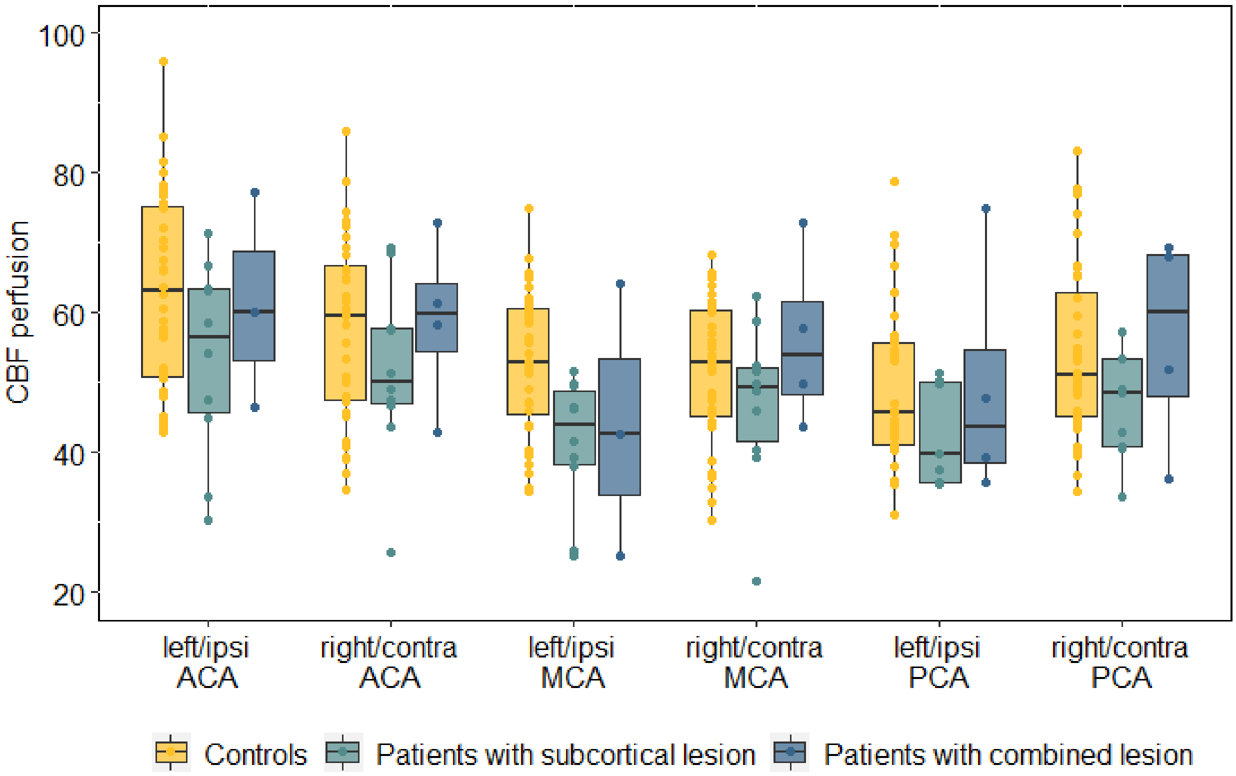

Note. Boxplots show median, interquartile range, minimum and maximum score and extreme values.

Figure S3. Cerebral blood flow imbalance in patients with subcortical lesions (n = 10), patients with combined lesions (n = 4) and controls.

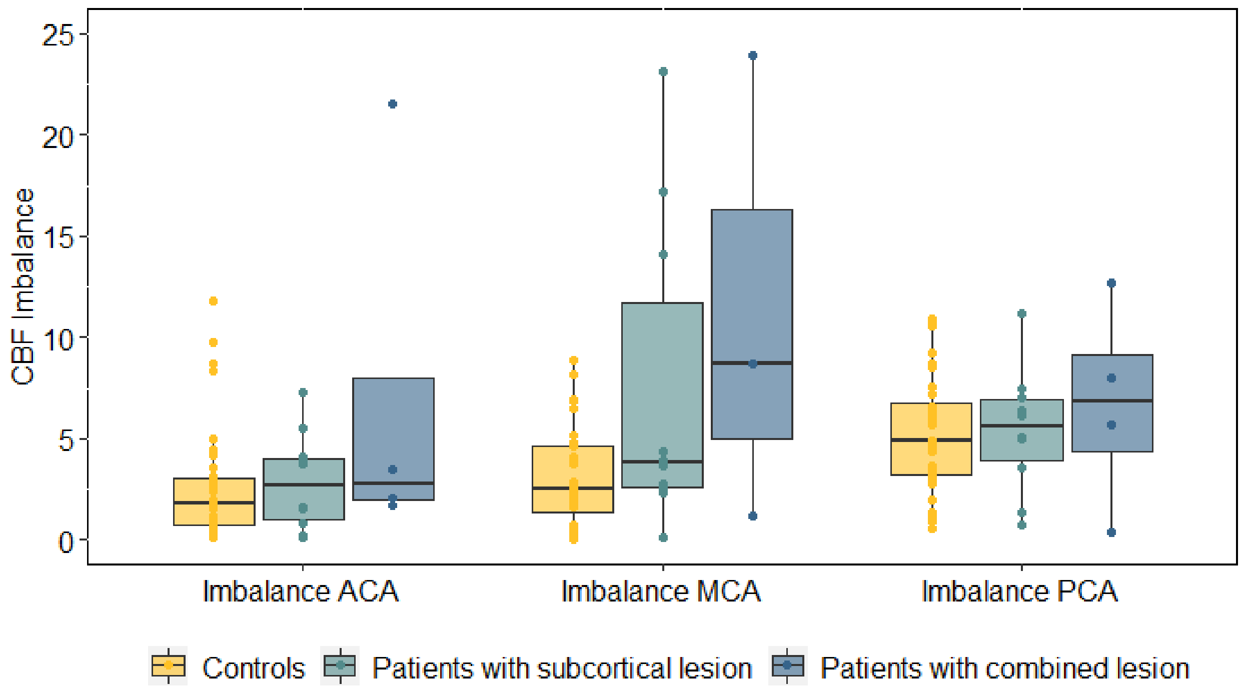

Note. Boxplots show median, interquartile range, minimum and maximum score and extreme values.
